# Two New Native β-Glucosidases from *Clavispora* NRRL Y-50464 Confer Its Dual Function as Cellobiose Fermenting Ethanologenic Yeast

**DOI:** 10.1371/journal.pone.0151293

**Published:** 2016-03-24

**Authors:** Xu Wang, Z. Lewis Liu, Scott A. Weber, Xiaoping Zhang

**Affiliations:** 1 Sichuan Agricultural University, Chengdu, China; 2 Bioenergy Research Unit, National Center for Agricultural Utilization Research, USDA-ARS, Peoria, Illinois, 61604, United States of America; National Renewable Energy Lab, UNITED STATES

## Abstract

Yeast strain *Clavispora* NRRL Y-50464 is able to produce cellulosic ethanol from lignocellulosic materials without addition of external β-glucosidase by simultaneous saccharification and fermentation. A β-glucosidase BGL1 protein from this strain was recently reported supporting its cellobiose utilization capability. Here, we report two additional new β-glucosidase genes encoding enzymes designated as BGL2 and BGL3 from strain NRRL Y-50464. Quantitative gene expression was analyzed and the gene function of *BGL2* and *BGL3* was confirmed by heterologous expression using cellobiose as a sole carbon source. Each gene was cloned and partially purified protein obtained separately for direct enzyme assay using varied substrates. Both proteins showed the highest specific activity at pH 5 and relatively strong affinity with a *K*_*m*_ of 0.08 and 0.18 mM for BGL2 and BGL3, respectively. The optimum temperature was found to be 50°C for BGL2 and 55°C for BGL3. Both proteins were able to hydrolyze 1,4 oligosaccharides evaluated in this study. They also showed a strong resistance to glucose product inhibition with a *K*_*i*_ of 61.97 and 38.33 mM for BGL2 and BGL3, respectively. While BGL3 was sensitive showing a significantly reduced activity to 4% ethanol, BGL2 demonstrated tolerance to ethanol. Its activity was enhanced in the presence of ethanol but reduced at concentrations greater than 16%. The presence of the fermentation inhibitors furfural and HMF did not affect the enzyme activity. Our results suggest that a β-glucosidase gene family exists in *Clavispora* NRRL Y-50464 with at least three members in this group that validate its cellobiose hydrolysis functions for lower-cost cellulosic ethanol production. Results of this study confirmed the cellobiose hydrolysis function of strain NRRL Y-50464, and further supported this dual functional yeast as a candidate for lower-cost cellulosic ethanol production and next-generation biocatalyst development in potential industrial applications.

## Introduction

Beta-glucosidase (β-D-glucoside, glucohydrolase, EC 3.2.1.21) is a key member of cellulase enzyme complex that hydrolyzes glycosidic bonds from glycosides and oligosaccharides. It cleaves β1, 4 bonds between two glucose molecules or glucose-substituted molecules such as cellobiose and other β-linked oligosaccharides with release of glucose. This group of enzymes is found universally in all kinds of living organisms with divergent functions. β-glucosidases from bacteria and fungi are mostly classified in glycosyl hydrolase (GH) family 3 based on the frequently updated Carbohydrate-Active enZymes Database (http://www.cazy.org) with 135 GH families [1–3]. Extracellular β-glucosidase produced by microbes are able to pass through cell wall easily that is desirable for saccharification and fermentation of cellulose [4–7]. These enzymes exhibited cellulose hydrolysis and transglycosylation activities for bioethanol produced from lignocellulosic biomass [8, 9]. Based on relative enzyme activity and affinity toward cellobiose and *p*-nitrophenyl-β-D-glucopyranoside (*p*NPG), β-glucosidases can be further classified into three groups: (1) *β*-glucosidases with a higher affinity for *p*NPG, (2) *β*-glucosidases with a higher affinity for cellobiose, and (3) *β*-glucosidases with similar affinities for both substrates [3, 10]. Representative structure of β-glucosidase was available and catalytic nucleophile and proton donor region were identified [11]. Modification of the N- and C-terminal was able to increase the stability of the enzyme [12]. And the increased thermal stability of the enzyme improved economic bioethanol production [13].

Since β-glucosidase plays a significant role in cellulose deconstruction, research on β-glucosidase has drawn increased attention in recent years with the emergence of a lignocellulosic biomass-based economy [14, 15]. For renewable cellulosic ethanol production from lignocellulosic materials, it is necessary to supply external β-glucosidase since most ethanologenic agents are unable to perform this terminal cellulolytic function in an economic simulteneous saccharification and fermentation (SSF) process. Conventional commercial cellulase products are capable of digesting high molecular weight cellulose but deficient in β-glucosidase. Thus the β-glucosidase activity is required to complete the enzymatic hydrolysis for cellulose-to-simple sugar conversion. Naturally occurring cellulolytic microorganisms often do not produce significant amount of ethanol. On the other hand, conventional ethanologenic agent is unable to produce sufficient β-glucosidase and an external source of β-glucosidase is required to complete the cellulose deconstruction for microbial fermentation. The high cost of enzyme is a major bottleneck for sustainable cellulosic ethanol production. For economic reasons, a practice using combined cellulolytic microorganisms and ethanologenic agent was also observed. However, the extra microorganism consumes additional carbon source and reduce the final product and fermentation efficiency. Recently, significant genetic engineering efforts have been taken to improve cellulolytic enzyme production and enable ethanologenic microbes to produce β-glucosidase [16]. For example, cellulolytic enzyme yield was significantly improved for a non-fermentation fungus *Neurospora crassa* by genetic engineering [17, 18]. *Saccharomyces cerevisiae* was enabled to utilize cellulose by integration of endoglucanase and β-glucosidase genes from *Tichoderma viride* [19]. *S*. *cerevisiae* was also able to produce cellulolytic enzymes by genetic engineering genes encoded for cellobiohydrolases from *Aspergillus aculeatus* and *T*. *reesei* [20]. However, significant challenges remains since the enzyme yield and the rate of conversion achieved so far are not yet satisfactory for potential industrial applications.

Recently, a fast-growing yeast strain of *Clavispora* NRRL Y-50464 was reported to produce cellulosic ethanol from corncobs and rice straw without addition of external β-glucosidase [21–23]. It produced 40.44 g/L ethanol from a pure cellulose within 72 h in a bottled SSF, and 32 g/L ethanol from corn stover solids at 48 h in a 2-liter bioreactor SSF using its native β-glucosidase [24]. Recent isolation and characterization of β-glucosidase BGL1 supported its enzyme production of *Clavispora* NRRL Y-50464 [25]. In this study, we report our findings of two new additional β-glucosidases, BGL2 and BGL3, from *Clavispora* NRRL Y-50464. These results suggested the presence of a β-glucosidase gene family in this yeast and further evidenced its dual function of cellobiose hydrolysis and fermenting capabilities for cellulosic ethanol production. New knowledge obtained from this research will aid development of next-generation biocatalysts for low-cost biofuel production in industrial applications.

## Materials and Methods

### Yeast, bacterial strain, media, and chemicals

Yeast strain *Clavispora* NRRL Y-50464 obtained from the Agricultural Research Service Patent Culture Collections (Peoria, IL, USA) was used in this study. Cell cultures were maintained and precultured using yeastpeptone (YP) medium containing 10 g yeast extract, 20 g peptone, and 50 g glucose in one liter distilled water. *Escherichia coli* TOP10 and *Pichia* expression and transformation kits from Invitrogen (Carlsbad, CA, USA) were applied for gene cloning and selection procedures. An YP medium amended with 5% cellobiose was used for gene expression assays. All oligosaccharides were purchased from Sigma-Aldrich (St. Louis, MO, USA) including cellobiose, cellotriose, cellotetraose, cellopentaose, laminaribiose, laminaritriose, laminaritetraose, laminaripentaose, laminarin, α-lactose, lichenan, salicin, and gentiobiose, and metal ions and chemicals KCl, CaCl_2_, ZnCl_2_, MgCl_2_, CuCl_2_, CoCl_2_, HgCl_2_, FeCl_2_, FeCl_3_, BaCl_2_, PbCl_2_, LiCl, NiCl_2_, MnCl_2_, SDS, triton X-100, 2-furaldehyde (furfural), and 5-(hydroxymethyl)-2-furaldehyde (HMF).

### Gene cloning

Standard procedures and molecular biology techniques were used in DNA manipulations as previously described [26]. Yeast genomic DNA from *Clavispora* NRRL Y-50464 was isolated using a DNeasy Blood & Tissue Kit (QIAGEN Sciences, Maryland, USA) following instructions from the manufacture. PCR reaction was performed using QIAGEN Taq PCR Master Mix Kit using 50 μl reaction volume with a profile as follows: 96°C for 3 min, 34 cycles of 96°C for 30 s, 59°C for 1.5 min, and 72°C for 2.5 min, and a final extension at 72°C for 10 min. Gene encoding regions for each target gene were amplified separately using primers incorporated with varied restriction enzyme digestion sites (Table 1). Currently, genome sequence of strain Y-50464 is not available. A related species *Clavispora lusitaniae* was reported to contain seven hypothetical proteins of β-glucosidase based on computation annotation (http://www.genome.jp/kegg/kegg.html). Using sequence of this genome as a reference, primers were designed for amplification of *BGL* fragments from strain Y-50464 using Primer3Plus (http://www.bioinformatics.nl/cgi-bin/primer3plus/primer3plusHelp.cgi). The DNA fragment was confirmed by sequence analysis using ABI 3730 DNA Analyzer. A verified DNA sample was recovered from an agarose gel using a QIAquick Gel Extraction kit (QIAGEN Sciences, Maryland, USA) and purified using a QIAquick PCR Purification Kit. The purified DNA fragment was then subcloned into *E*. *coli* DH5α using an Invitrogen TOPO TA Cloning Kit. Positive clones were selected on a medium containing 50 μg/ml ampicillin and verified by restriction digest and sequence analysis. A recombinant plasmid was constructed using *Pichia* expression vectors pPICZ A or B containing a tightly regulated *AOX1* promoter and zeocin resistance gene (Invitrogen, Carlsbad, CA, USA). Targeted *Pichia* transformants were selected on a medium containing 100 μg/ml zeocin, and protein expression was tested using Invitrogen *Pichia* X33 strain containing a polyhistidine tag to a C-terminal peptide.

**Table 1:** Primers used in this study with incorporated endonuclease restriction sites underlined.

ID	Sequence (5’-3’)
BGL2_F	TCAGTTCACTGGTACCATACTAGAAAAAGTAAACTTGACCACAGGGAC
BGL2_R	AGTCAAGAGCTCCCCACTAAGATCAATCTCACCATAGAGCTC
BGL3_F	TGGCTGACCACGTGATTGACGTGGAAAA
BGL3_R	ACCTTGATCTAGAATTCTTGGATCAACTCAACG
qBGL2_F	GTTTTGCTTCGGGACCTTGT
qBGL2_R	TGAATGTACTACCGGTGGCT
qBGL3_F	CATTGTGGACGAGTTGTTCT
qBGL3_R	CTTGTTGTCGATCACCAACT

### Heterologous expression of gene clone

Targeted clones pPICZαA-*BGL2* and pPICZαB-*BGL3* from *Clavispora* NRRL Y-50464 were transformed into the *Pichia* expression strain in YP medium containing 5% cellobiose and 100 μg/ml zeocin. *Clavispora* NRRL Y-50464 strain was used as a positive control and the *Pichia* vector of plasmid pPICZαB without a gene insert served as a negative control. All strains were precultured on a 2% glucose medium with agitation at 250 rpm at 30°C overnight. Then cells were washed and resuspended in a medium in a 250 ml flask containing 5% cellobiose. Cells were incubated under the same conditions and growth monitored by OD_600_.

### Performance of cellobiose fermentation and clone selection

The *Pichia* transformed strains with *BGL2* or *BGL3* gene insert were evaluated for ethanol production using cellobiose as a sole carbon source. The freshly prepared strains were transferred into yeast-peptone (YP) medium containing 5% cellobiose and incubated at 30°C for 20 h. The wild type strain NRRL Y-50464 served as a positive control and the vector without a gene insert served as a negative control. Two replications were carried out for each strain. Ethanol production for each strain was measured by HPLC as previously described [23]. One clone strain with the highest ethanol concentration was selected from each gene group for further study.

### Gene expression

Quantitative gene expression was analyzed for each gene in recombinant *Pichia* strains. Cells were prepared and incubated using the same procedures as described above. The end of cultivation on glucose was recorded as 0 h and cell samples taken as background measurement. Cells were then washed and transferred on to a cellobiose medium and samples were taken at 4, 8, 20, and 26 h after growth. To preserve the fidelity of targeted mRNA, cells were harvested within 2 min without refrigeration and cell pellet frozen immediately into dry ice until use. DNA was extracted following previously described procedures [27, 28]. Briefly, frozen cells were resuspended in 500 μl of pre-warmed acid phenol:chlorofonn solution at 65°C and 500 μl of TES buffer (10 mM Tris pH7.5, 10 mM EDTA, and 0.5% SDS) added immediately. The mix was vortexed vigorously and incubated at 65°C and RNA extracted by centrifugation for 20 min at 3200 xg at 4°C. The RNA was purified using phenol:chlorofonn (pH 4.7) and precipitated using sodium acetate and cooled ethanol. The purified RNA was dissolved in 200 μl of RNA storage buffer (Ambion, Inc. Austin, TX) and stored at—80°C until use. RNA yield, integrity, and purity were measured by denatured gel electrophoresis and spectrophotometry. Samples with RNA purity greater than 1.8 were used. A set of external mRNA was employed in the qRT-PCR assays and a master equation applied for data normalization and analysis as previously described [27]. The qRT-PCR was performed in 25 μl reaction volume using SYBR Green FAST Mastermix from QIAGEN on Applied Biosystem 7500 Real Time PCR System. The PCR profile was defined as follows: 95°C initial degradation for 3 min, 40 cycles of 95°C for 15 s, 60°C for 45 s (stat collection), 95°C for 15 s, 60°C for 1 min, 95°C for 15s, for the dissociation curve, 95°C for 15 s, 60°C for 1 min, 95°C for 15 s, and held at 4°C after reaction. Two biological experiments were carried out with three technical replications for each experiment.

### Protein isolation and purification

Five ml of cultural solution withfresh cells grown on a glucose medium were collected when OD_600_ reached 1.0 and transferred into 500 ml YP medium containing 5% cellobiose and 100 μg/ml zeocin similar as described above. The culture was incubated at 30°C with 250 rpm agitation for 24 h. Cells were harvested and lysed using Y-PER Plus from Thermo Scientific (Rockford, IL, USA) following manufacturer instructions. The homogenate was centrifuged at 20,000×g for 30min and the pellet was discarded. The supernatant fraction was applied to a column (1.5×1.0cm) of Ni-NTA equilibrated with 50 mM sodium phosphate binding buffer (pH 7.5). Protein sample was cleaned using a washing buffer (binding buffer plus 300 mM NaCl). The expressed protein was eluted by applying a step-wise gradient of 50, 100, 150, and 200 mM imidazole in the binding buffer. Protein samples were kept on ice and processed within 24 h. Molecular weight of the purified protein was estimated by SDS-PAGE with a 12% resolving and 5% stacking gel. The partially purified protein was stored at 4°C and used for enzyme activity assays within 48 h.

### Enzyme activity assays

Enzyme assay was performed using commonly applied *p*-nitrophenyl-β-D-glucopyranoside (*p*NPG) method as previously described [25, 29, 30]. Protein concentration was calculated using a Genesys 10UV spectrophotometer (Thermo Scientific, West Palm Beach, FL, USA) following a previously described procedure (http://www.strgen.org/protocols). Concentration of protein was estimated as 0.2935 and 0.8066 mg/ml for BGL2 and BGL3, respectively. Each assay was carried out in a total volume of 125 μl reaction mixture. Fifteen μl of partially purified protein sample was added into 110 μl of 100 mM citrate buffer with 5 mM substrate. The protein samples were kept on ice and all reagents were maintained in an incubator under designated temperature. The reaction was carried out in an incubator for 30 min. Then 125 μl ice cold 0.5 M Na_2_CO_3_ was added into each well to stop the reaction. The optimum pH for specific enzyme activity was determined at pH levels ranging from 3.0 to 8.0 and optimum temperature was determined by performing the assay in the range of 30 to 80°C. Reactions in the 96-well plate were measured immediately using a Power Wavex 340 plate reader at 405 nm (Bio-Tek Instruments Inc., Winooski, VT, USA).

### Substrate specificity to oligosaccharides

In addition to assay using the chromogenic substrate *p*NPG, specific activity of the partially purified proteins toward selected oligosaccharides was evaluated, including cellobiose, cellotriose, cellotetraose, cellopentose, laminaribiose, laminaritriose, laminaritetraose, laminaripentaose, laminarin, lactose, lichenan, gentiobiose, salicin, and sophorose. Cellobiose was used as a control and relative activity recorded for each substrate. Experiments were carried out in triplicate.

### Evaluation of inhibition by glucose and ethanol

To evaluate inhibiting effect of glucose and ethanol, serial concentrations of glucose ranging from 0 M to 2 M were used to add in the reaction mixture separately during the enzyme assay. Varied concentrations of ethanol from 0 to 32% were used to examine the effect of ethanol on the enzyme activity. Inhibition was measured by specific activity relative to a reference reaction without addition of glucose or ethanol as previously described [31]. Each assay was carried out in a total volume of 125 μl reaction mixture. A 15 μl purified protein sample was added into 110 μl of 100 mM citrate buffer with 5 mM *p*NPG and a specific concentration of glucose or ethanol. The final concentration of glucose in the reaction mix was 0, 50, 100, 200, 400, 600, 800, 1000 and 2000 mM, respectively. Each reaction against ethanol was performed at a concentration of 0, 4, 8, 16, 20, 24, 28 and 32 percent separately. Assays were carried out in triplicate under optimal pH and temperature conditions.

### Inhibition by metal ions and Chemicals

Effects of selective metal ions and chemicals on β-glucosidase activity were also evaluated. Fifteen metal ions were used at a final concentration of 1 mM applied for each cation, including K^+^, Na^+^, Li^+^, Ca^2+^, Mg^2+^, Pb^2+^, Zn^2+^, Mn^2+^, Cu^2+^, Fe^2+^, Ni^2+^, Co^2+^, Ba^2+^, Hg^2+^ and Fe^3+^ (all supplied as chloride forms). For other chemicals, ethylenediaminetetraacetic acid (EDTA), sodium dodecyl sulfate (SDS), triton X-100, furfural and HMF, a 10 mM of each chemical was applied separately to the reaction mixtures. Each assay was performed in a total volume of 125 μl reaction mixture. A purified protein sample of 15 μl was added into 110 μl of 100 mM citrate buffer with 5 mM *p*NPG and 1 mM metal ion or 10 mM chemical. Then the reaction mix was placed in an incubator for 30 min, followed by addition of 125 μl ice cold 0.5 M Na_2_CO_3_ to stop the reaction. A reaction without addition of a metal ion or chemical served as a control. Residual activity was calculated as described above. Each assay was performed in triplicate.

### Sequence analysis

Amino acid sequences of BGL1, BGL2, and BGL3 from *Clavispora* NRRL Y-50464 were analyzed using Biological Workbench v3.2 (http://workbench.sdsc.edu/) in comparison with BGL2 from *Trichoderma harzianum* (NCBI accession no. EF426299, ThBGL2) [32], BGL from *Trichoderma reesei* (Uniprot accession no. O93785, TrBGL) [33], BGL1 from *Kluyveromyces marxianus* (GenBankTM accession number ACY95404.1, KmBGL1) [34], BGL1 from *Aspergillus niger* (GenBankTM accession number AJ132386.1, AnBGL1)[35], BGL3a from *Myceliophthora thermophila* (Model ID 66804; chromosome 3:4861135–4863642, DOE Joint Genome Institute, MtBGL3a) [36], and BGLb from *Bacillus polymyxa* (GenBankTM accession number M60211, BpBGLb) [37]. A phylogenetic tree was constructed to access relationships among these β-glucosidases with additional BGLs from *Neurospora crassa* (Ncbgl1, Ncbgl2, Ncbgl4, and Ncbgl6) using the neighbor-joining method in the MEGA 5.10 [38].

## Results

### Gene clone and heterologous expression of *BGL2* and *BGL3*

**Fig 1:**
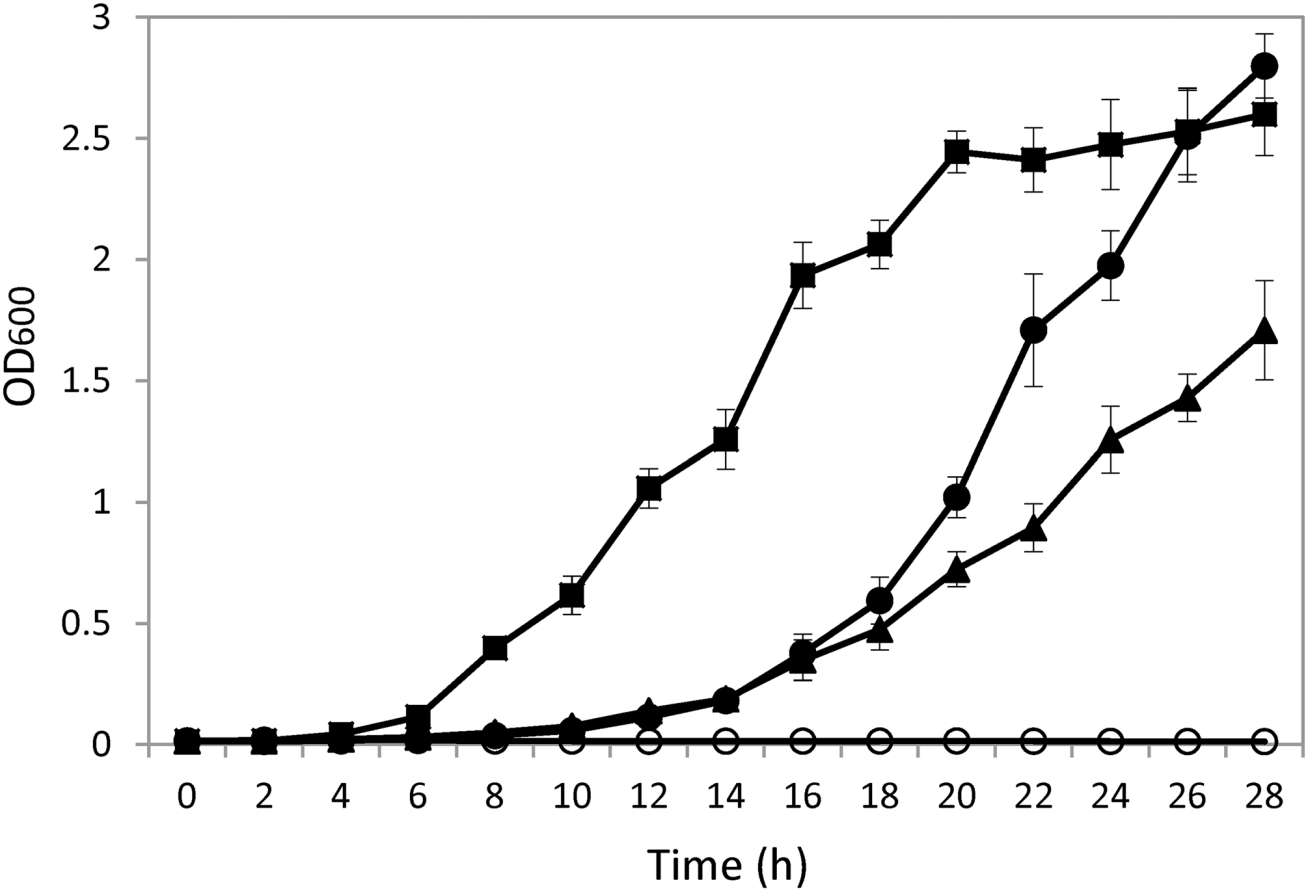
Comparison of cell growth on a medium containing 5% cellobiose and 100 μg/ml zeocin for strain *Clavispora* NRRL Y-50464 (■), clone pPICZαA-*BGL2* (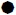), pPICZαB-*BGL3* (▲), and an empty vector without a gene insert (◯).

Genes of *BGL2* and *BGL3* from *Clavispora* NRRL Y-50464 were cloned into a plasmid pPICZαA and pPICZαB separately resulting in pPICZαA-*BGL2* and pPICZαB-*BGL3*, respectively. The obtained clones were verified by sequencing analysis. Nucleotide sequences and deduced amino acid sequences of *Clavispora BGL2* and *BGL3* were deposited at NCBI GenBank with accession numbers KR011270 and KR011269, respectively. Both genes were successfully overexpressed in the Invitrogen X33 *Pichia* strain. On a medium containing 5% cellobiose and 100 μg/ml zeocin, both strains were able to utilize cellobiose and cell growth reached a stationary phase as early as 28 h (Fig 1). Growth rate of the *BGL2* containing strain was significantly faster than that of *BGL3*. In contrast, a vector without the targeted gene insert was unable to grow on the cellobiose medium (Fig 1). A wild type of strain NRRL Y-50464 was also included for comparison, which showed significantly faster growth reaching the stationary phase earlier at 20 h. These results confirmed the function of the two newly cloned genes *BGL2* and *BGL3* that encode β-glucosidase enzyme activity in hydrolyzing and utilizing cellobiose.

### Clone selection based on ethanol production from cellobiose

**Fig 2:**
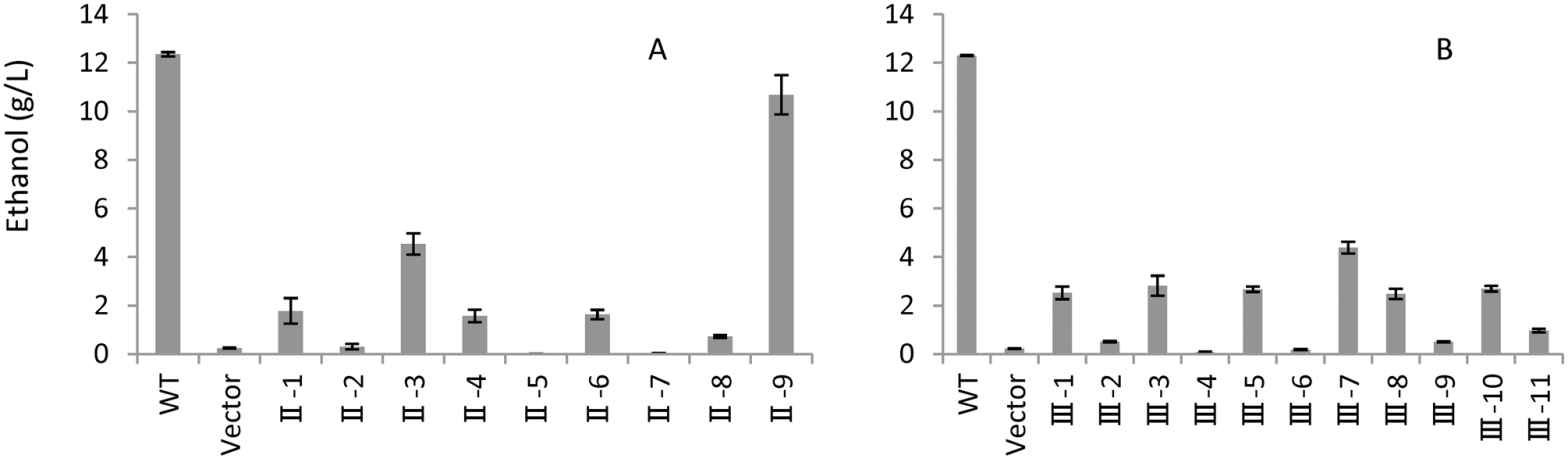
Ethanol production of gene clones using cellobiose as a sole carbon source for strains representing *BGL2* (A) and *BGL3* (B) 20 h after incubation. Strain NRRL Y-50464 (WT) served as a positive control and a vector without a gene insert served as a negative control. Values are means of two replications.

Among nine clones examined for ethanol production from cellobiose as sole carbon source, clone II-9 produced 9.75 g/L ethanol, the highest ethanol concentration observed, and was selected for characterization of *BGL2* (Fig 2A). Clone III-7 with a relatively higher ethanol production of 3.21 g/L was selected from 11 strains tested as a representative of *BGL3* (Fig 2B).

### Gene expression

**Fig 3:**
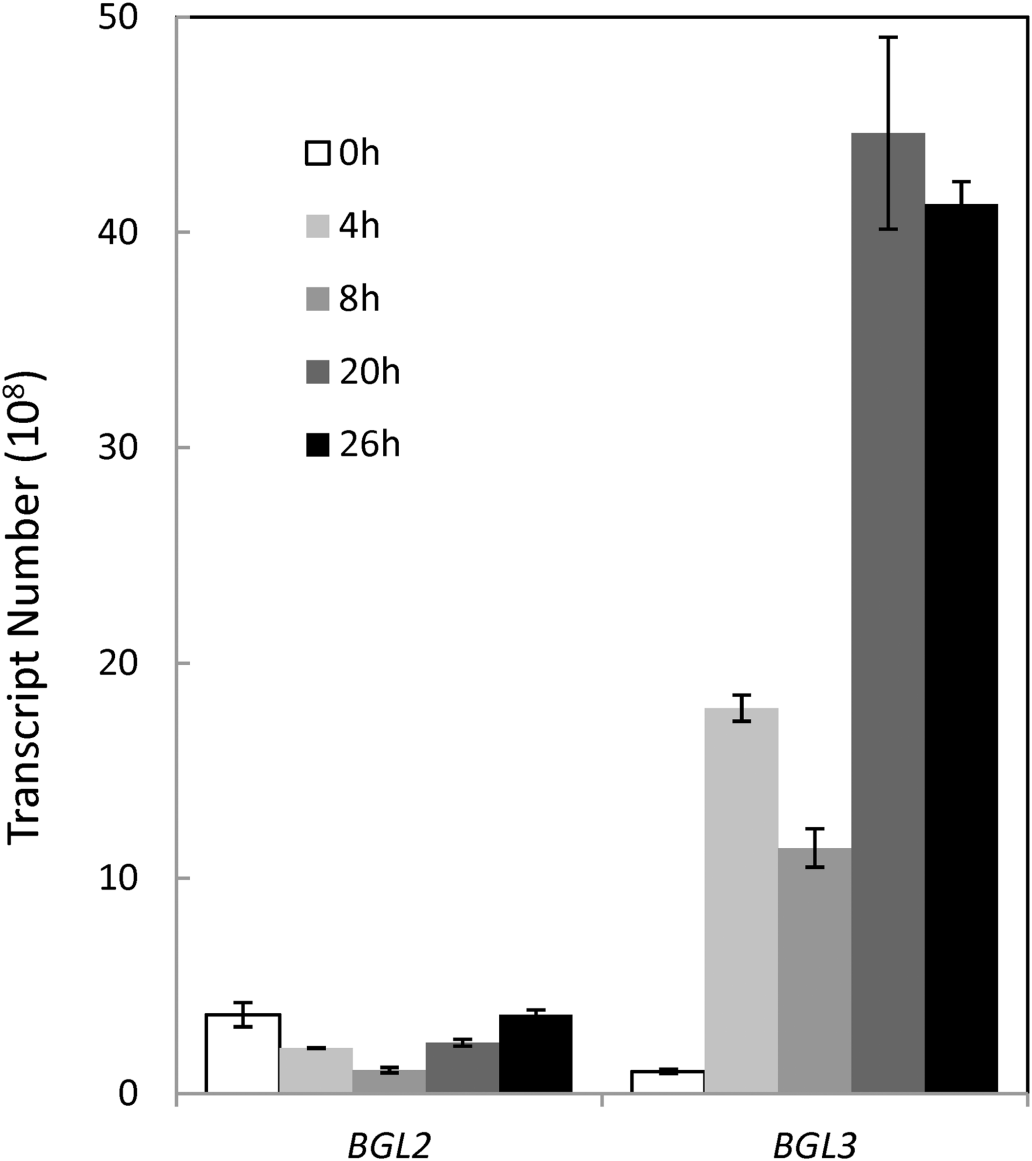
Quantitative gene expression of *BGL2* and *BGL3* from *Clavispora* NRRL Y-50464 on a medium containing 5% cellobiose and 100 μg/ml zeocin as measured by mRNA abundance for cells grown on cellobiose at 0, 4, 8, 20, and 26 h. Values are means of two replications.

Quantitative gene expression each for *BGL2* and *BGL3* was examined using cells grown on glucose or cellobiose separately. On a medium containing glucose (shown as 0 h), higher levels of mRNA transcripts at 4 x 10^8^ and 1 x 10^8^ copies were observed for *BGL2* and *BGL3*, respectively (Fig 3). The initial background expression level of *BGL2* on glucose was significantly higher than that of *BGL3*. When shifted to cellobiose, transcript of *BGL3* showed an increase of approximately 15-, 9-, 38-, and 35-fold increase over 4, 8, 20, and 26 h period of time, respectively (Fig 3). For *BGL2*, the number of transcript was slightly decreased when the sugar was shifted from glucose to cellobiose but gradually recovered over time. This was unexpected since the *BGL2* expressing strain displayed a significantly higher rate of cell growth on cellobiose medium and a higher level of specific activity.

### Protein isolation and purification

**Fig 4:**
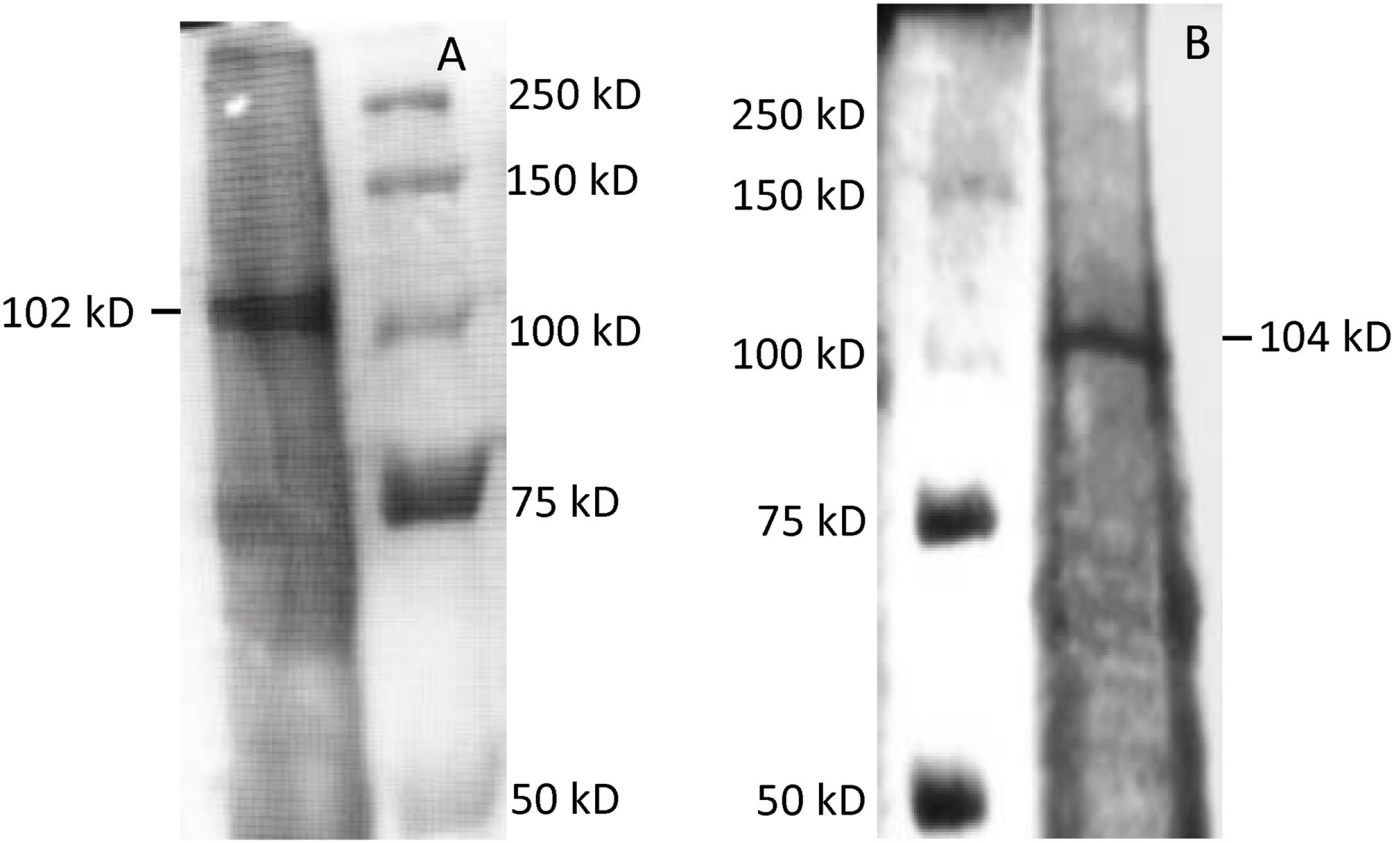
SDS-PAGE gels showing partially purified β-glucosidase BGL2 (A) and BGL3 (B) from *Clavispora* NRRL Y-50464 with an estimated molecular size at 102.7 and 104.8 kD, respectively. The net molecular weight of deduced amino acid of BGL2 and BGL3 was estimated approximately at 88.3 and 92.5 kD, respectively.

A partially purified protein from each gene clone was obtained using Ni-NTA column chromatography with a polyhistidine-tag. The partially purified BGL2 and BGL3 protein showed molecular masses of approximately 102.7 and 104.8 kD, respectively on a SDS-PAGE gel (Fig 4). The deduced amino acid sequence of BGL2 and BGL3 contain 804 and 837 residues, respectively. The net molecular weight of BGL2 was estimated at approximately 88.3 kD and BGL3, 92.5 kD. SDS-PAGE gels showing crude protein elusions for BGL2 and BGL3 were presented in S1 Fig.

### Enzyme kinetics and optimal temperature and pH

**Fig 5:**
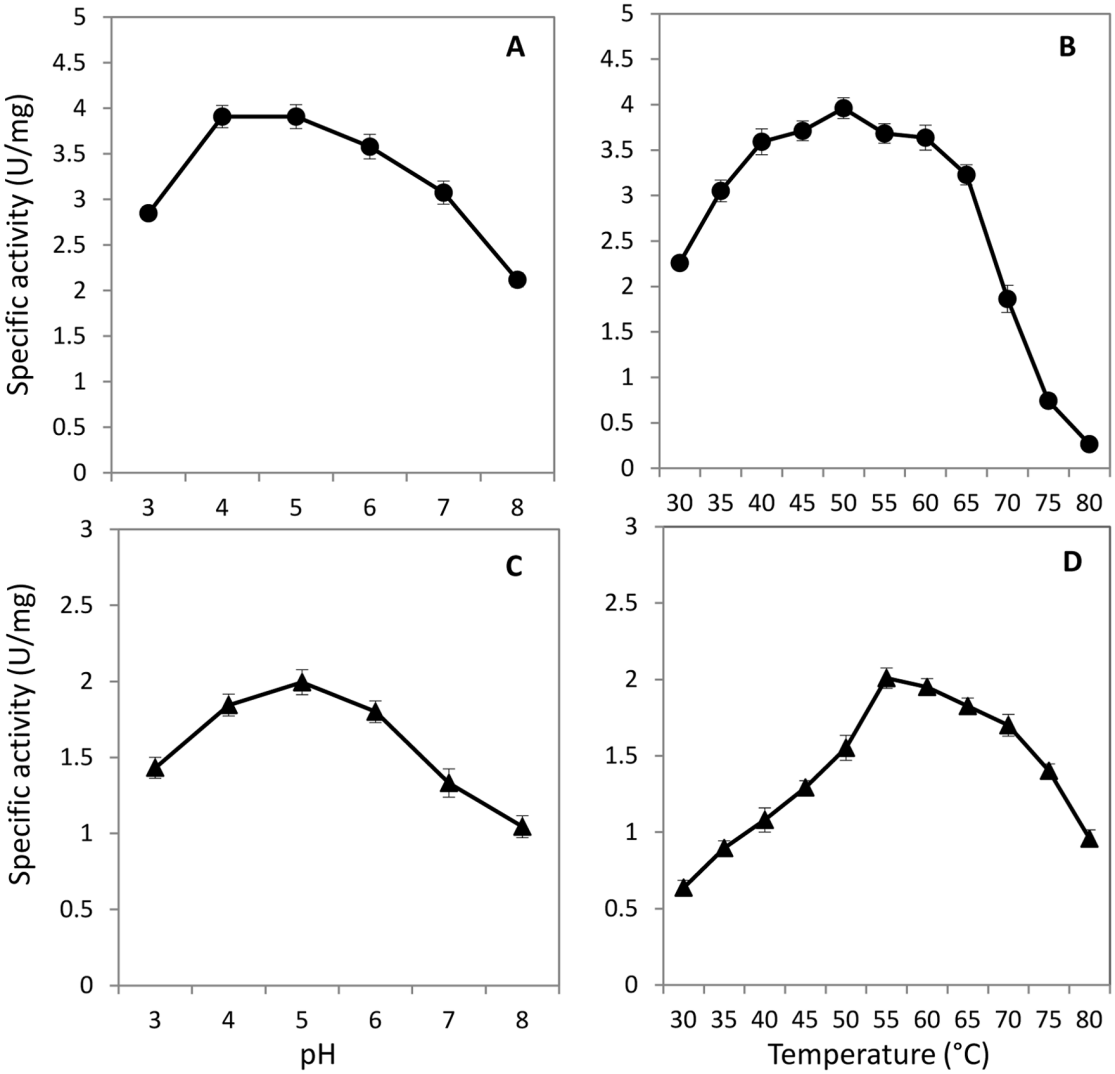
Specific activity of partially purified β-glucosidase BGL2 (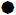) and BGL3 (▲) from *Clavispora* NRRL Y-50464 showing activity ranges of pH (A and C) and temperature (B and D). Values are means of three replications.

The partially purified protein of BGL2 showed a higher level of enzyme specific activity with a lower *K*_*m*_ of 0.08 mM when compared with that of BGL3 with a *Km* of 0.18 mM (Table 2). The enzyme activity of both proteins appeared to be superior to the previously reported BGL1 as measured by their *K*_*m*_. Other kinetic parameters for each enzyme were also provided in Table 2. BGL2 was active over a wide range of temperatures from 40 to 60°C with the highest activity observed at 50°C (Fig 5B). Similarly higher activity was observed at pH 4 and 5 with the highest activity recorded at pH 5 (Fig 5A). In contrast, BGL3 appeared to prefer a higher temperature with its highest specific activity recorded at 55°C (Fig 5D). Its activity remained relatively higher from 60 to 70°C. The optimal pH for BGL3 was at 5 (Fig 5C).

**Table 2:** Kinetic parameters of a partially purified protein of BGL2 or BGL3 from *Clavispora* NRRL Y-50464 using *p*NPG as a substrate.

Protein	*Vmax (μmol min-1 mg-1)*	*Kcat (min-1)*	*Kcat/Km (mM-1 min-1)*	*Km (mM)*	*K i (mM) (against glucose)*	Reference
BGL2	*5*.*27 ± 0*.*11*	*547*.*89 ± 15*.*91*	*6,834*.*23 ± 94*.*47*	*0*.*08 ± 0*.*01*	*61*.*97±2*.*49*	This work
BGL3	*1*.*63 ± 0*.*04*	*84*.*04 ± 1*.*06*	*462*.*50 ± 14*.*12*	*0*.*18 ± 0*.*02*	*38*.*33±1*.*15*	This work
BGL1	*5*.*91*	na*	*na*	*0*.*355*	*15*.*2*	[11]
Novo188	*4*.*2*	*na*	*na*	*0*.*448*	*0*.*735*	[11]

*na: not available. Data from this work are presented by mean values with standard deviations (n = 3).

### Inhibition effect of glucose and ethanol

**Fig 6:**
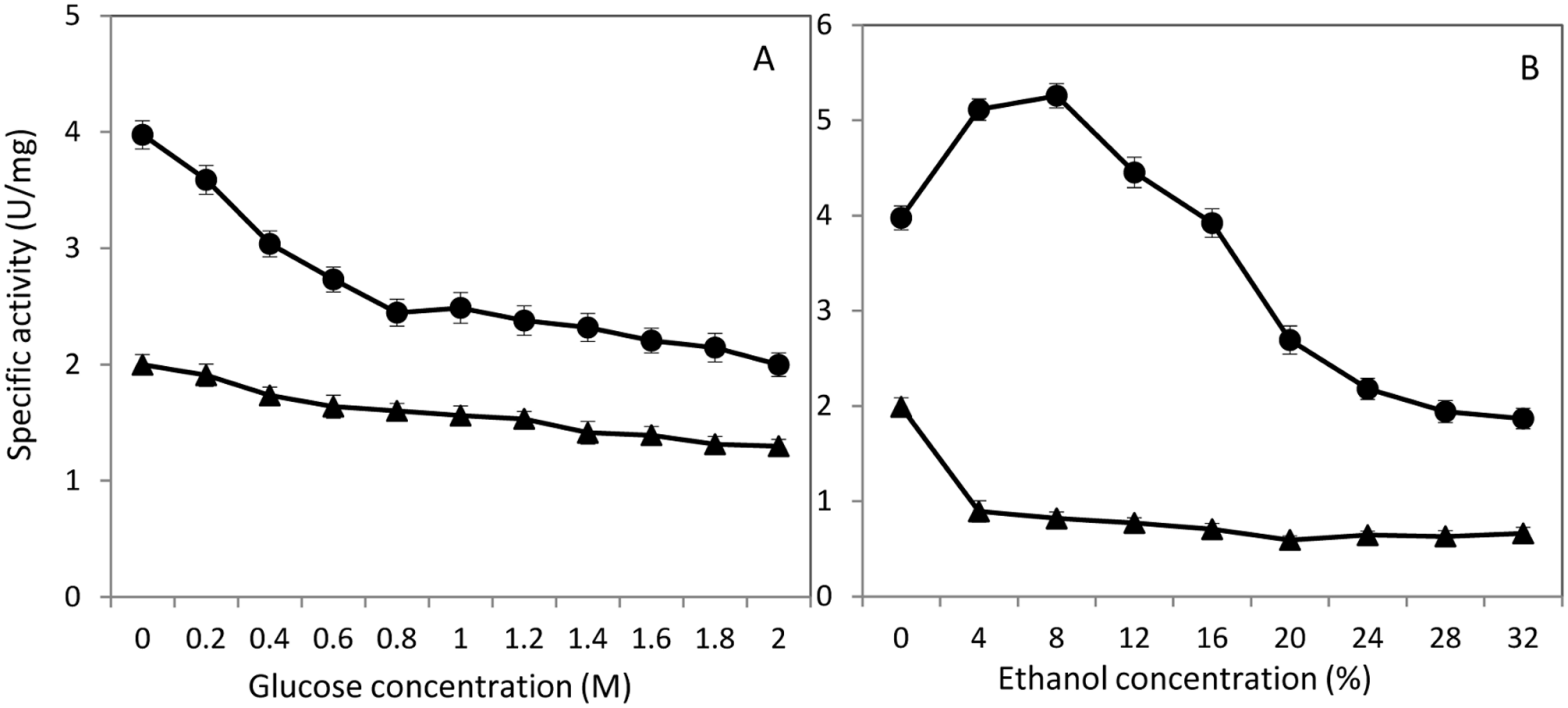
Specific activity of partially purified β-glucosidase BGL2 (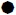) and BGL3 (▲) from *Clavispora* NRRL Y-50464 showing inhibition effect by increased concentrations of glucose (A) and ethanol (B). Values are means of three replications.

Product inhibition of β-glucosidase by glucose is a practical concern affecting the efficiency of the enzyme activity. Since the target application of the enzyme is in cellulosic ethanol production, we are also interested in the impact of ethanol production to the performance of β-glucosidase in a consolidated bioprocessing procedure. In this study, we did not observe significant inhibition by glucose for both proteins. BGL2 and BGL3 showed high levels of tolerance to glucose with a *Ki* of 61.97 and 38.33 mM, respectively (Table 2). They were superior to the previously reported BGL1. Both proteins did not show significant reduced activities until glucose concentration increased to 400 mM (Fig 6A). Although BGL3 appeared to have a relatively lower activity, it was more tolerant than BGL2 even at extremely high concentrations of glucose. However, BGL3 was highly susceptible to challenge by ethanol showing a significant inhibition starting at a lower concentration of 4% (Fig 6B). For BGL2, we observed its activity to be enhanced with the addition of ethanol at lower concentrations from 4 to 8%. It appeared BGL2 tolerated ethanol up to 16% and its activity declined at 20% and higher concentrations.

### Substrate specificity toward oligosaccharides

Since diversified oligos exist in the complex cellulosic materials, we evaluated enzyme activity toward 14 oligosaccharides as a substrate, including cellobiose, cellotriose, cellotetraose, cellopentose, laminaribiose, laminaritriose, laminaritetraose, laminaripentaose, laminarin, lactose, lichenan, gentiobiose, salicin, and sophorose. In general, BGL2 showed variable hydrolytic activity at moderate levels toward all examined substrates with the lowest activity of about 50% against laminarin, compared to that of cellobiose (Table 3). On the other hand, BGL3 displayed stronger hydrolytic activity toward most oligosaccharides when compared with cellobiose. Even for laminarin, it displayed almost 80% relative activity. However, the overall specific activity of BGL3 for all substrates tested was lower than that of BGL2.

**Table 3:** Specific activity of partially purified BGL2 and BGL3 from *Clavispora* NRRL Y-50464 towards selective substrate of oligosaccharides.

Oligosaccharides	Linkage of glycosyl group	BGL2		BGL3	
		Specific activity (U/mg)	Relative activity (%)	Specific activity (U/mg)	Relative activity (%)
Cellobiose	β (1,4)-Glucose	1.74 ± 0.06	100	0.66 ± 0.02	100
Cellotriose	β (1,4)-Glucose	1.35 ± 0.04	77.59	0.80 ± 0.02	121.21
Cellotetraose	β (1,4)-Glucose	1.14 ± 0.03	65.52	0.62 ± 0.01	93.94
Cellopentaose	β (1,4)-Glucose	1.53 ± 0.04	87.93	0.81 ± 0.02	122.73
Laminaribiose	β (1,3)-Glucose	1.56 ± 0.06	89.66	0.82 ± 0.01	124.24
Laminaritriose	β (1,3)-Glucose	1.28 ± 0.04	73.56	0.78 ± 0.02	118.18
Laminaritetraose	β (1,3)-Glucose	1.33 ± 0.04	76.44	0.76 ± 0.02	115.15
Laminaripentaose	β (1,3)-Glucose	1.33 ± 0.04	76.44	0.76 ± 0.02	115.15
Laminarin	β (1,3)-Glucose	0.91 ± 0.03	52.30	0.52 ± 0.01	78.79
Lactose	β 1-galactose, α 4-glucose	1.14 ± 0.04	65.52	0.71 ± 0.01	107.58
Lichenan	β (1,3–1,4)-Glucose	1.40 ± 0.06	80.46	0.73 ± 0.01	110.61
Gentiobiose	β (1,6)-Glucose	1.03 ± 0.03	59.20	0.73 ± 0.01	110.61
Salicin	β-Glucose	1.17 ± 0.03	67.24	0.69 ± 0.01	104.55
Sophorose	β (1,2)-Glucose	1.26 ± 0.03	72.41	0.75 ± 0.01	113.64

### Effect of metal ions and chemicals

During lignocellulosic biomass conversion to fuels and chemicals, numerous chemicals and metal ions are generated from biomass and mechanical sources during the biomass pretreatment procedure. Effect of these chemicals to enzyme performance is another concern in a consolidated bioprocessing process. Thus, we examined the effect of 15 metal ions and five chemicals including two well-know biomass fermentation inhibitors furfural and HMF on our recombinant BGLs. In this study, only copper showed inhibition effect to both proteins (Table 4). Most metals examined did not appear to be harmful for the enzyme hydrolysis activity and some metals enhanced the reaction at the concentrations examined. For example, Na^+^ and K^+^ enhanced BGL2 activity significantly and Fe^2+^ showed a very high level of enhancement to both BGL2 and BGL3. Furfural and HMF did not inhibit either enzyme. In fact, furfural enhanced the reaction of both enzymes. Since furfural is an unstable compound, so whether this stimulation is due to furfural directly or its reduced product is unknown.

**Table 4:** Effects of metal ions and chemicals on specific activity of partially purified BGL2 and BGL3 from *Clavispora* NRRL Y-50464 using *p*NPG as a substrate.

Treatment	BGL2		BGL3	
	Specific activity (U/mg)	Relative activity (%)	Specific activity (U/mg)	Relative activity (%)
Control	3.95 ± 0.07	100	1.96 ± 0.02	100
NaCl (1 mM)	6.86 ± 0.12	173.67	2.01 ± 0.03	102.55
KCl (1 mM)	6.76 ± 0.13	171.14	2.08 ± 0.03	106.12
CaCl_2_ (1 mM)	3.86 ± 0.09	97.72	2.13 ± 0.02	108.67
ZnCl_2_ (1 mM)	4.12 ± 0.10	104.3	2.68 ± 0.03	136.73
MgCl_2_ (1 mM)	4 ± 0.08	101.26	2.05 ± 0.02	104.59
CuCl2 (1 mM)	2.96 ± 0.03	74.94	1.67 ± 0.01	85.2
CoCl_2_ (1 mM)	4.15 ± 0.11	105.06	2.53 ± 0.03	129.08
HgCl_2_ (1 mM)	3.49 ± 0.08	88.35	2.37 ± 0.02	120.92
FeCl_2_ (1 mM)	11.31 ± 1.02	286.33	6.88 ± 0.76	351.02
FeCl_3_ (1 mM)	4.89 ± 0.10	123.8	2.46 ± 0.02	125.51
BaCl_2_ (1 mM)	3.88 ± 0.08	98.23	2.21 ± 0.01	112.76
PbCl_2_ (1 mM)	4.2 ± 0.11	106.33	2.27 ± 0.02	115.82
LiCl (1 mM)	4.01 ± 0.10	101.52	2.31 ± 0.03	117.86
NiCl_2_ (1 mM)	4.12 ± 0.09	104.3	1.95 ± 0.03	99.49
MnCl_2_ (1 mM)	4.1 ± 0.11	103.8	2.54 ± 0.02	129.59
EDTA (10 mM)	2.59 ± 0.12	65.57	2.13 ± 0.04	108.67

### Sequence analysis and relationships of β-glucosidases 1, 2, and 3

**Fig 7:**
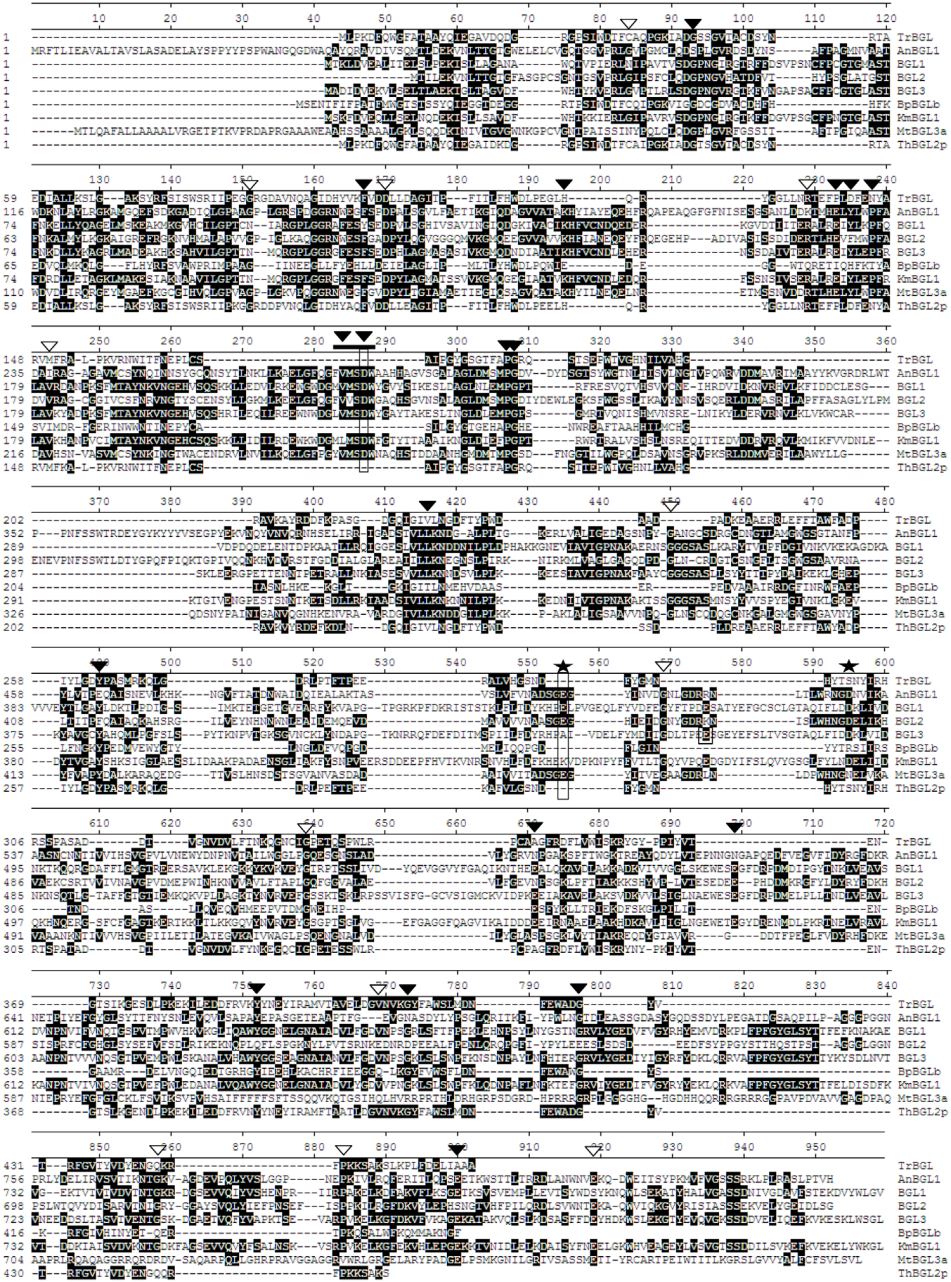
Comparison of amino acid sequences of BGL1, BGL2, and BGL3 from *Clavispora* NRRL Y-50464 with β-glucosidases from other species of *Aspergillus niger* (AnBGL1), *Trichoderma harzianum* (ThBGL2), *Trichoderma reesei* (TrBGL), *Kluyveromyces marxianus* (KmBGL1), *Myceliophthora thermophila* (MtBGL3a), and *Bacillus polymyxa* (BpBGLb). Matched consensus sequences are shaded in black with a white font. Strongly conserved sequence and weak conserved sequence is marked with a solid and open triangle, respectively. A conserved motif of FVMSDW for glycosyl hydrolase family 3 is indicated by a bold line. Conserved acidic residue found in *A*. *niger* is marked with a star. Important residues of catalytic nucleophile located at Asp225, Asp224, and Asp225 the N-terminal domain and the proton donor Glu449, Glu459, and Glu459 in the C-terminal domain were boxed respectively for the three BGLs from strain Y-50464.

Amino acid sequence alignment of β-glucosidases from strain Y-50464 and other six species showed many conserved sequences in common (Fig 7). A conserved motif of FVMSDW commonly found for GH family 3 members was observed for all fungal BGLs, except for the bacterial BGLb form *Bacillus polymyxa*. Varghese et al. identified Asp285 in the N-terminal domain as a catalytic nucleophile and Glu491 in the C-terminal domain as the proton donor in BGL of GH 3 from barley [11]. These locations were found to be at Asp282 and Glu511 for BGL1 from *Aspergillus niger* [35]. For BGLs from strain Y-50464 in this study, we found these important residues to be located at Asp225, Asp224, and Asp225; and Glu449, Glu458, and Glu459 for BGL1, BGL2, and BGL3, respectively (Fig 7). Most of them matched with the same position of AnBGL1 and other fungal BGLs. A few conserved acidic catalyst reported from AnBGL1 in *A*. *niger* [39] were also found in BGLs from strain Y-50464.

**Fig 8:**
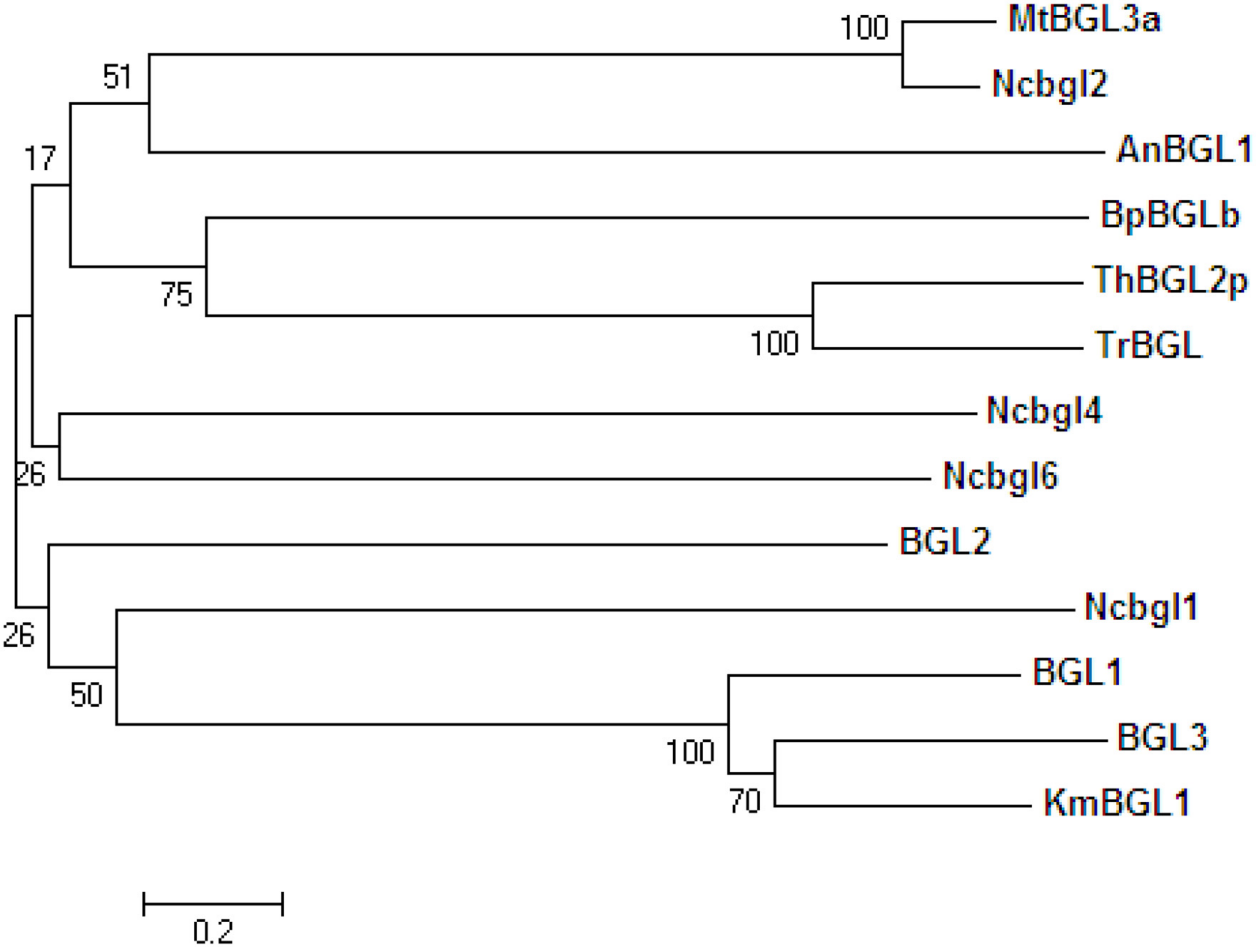
A phylogenetic tree derived from amino acid sequences analysis showing relationships of β-glucosidases from *Clavispora* NRRL Y-50464 (BGL1, BGL2, and BGL3) with other microbial BGLs from *Aspergillus niger* (AnBGL1), *Trichoderma harzianum* (ThBGL2), *Trichoderma reesei* (TrBGL), *Kluyveromyces marxianus* (KmBGL1), *Myceliophthora thermophila* (MtBGL3a), *Neurospora crassa* (Ncbgl1, Ncbgl2, Ncbgl4, and Ncbgl6), and *Bacillus polymyxa* (BpBGLb) using the neighbor-joining method in the MEGA 5.10.

Phylogenetic analysis of 13 microbial BGLs showed that BGL1, BGL2 and BGL3 from strain Y-50464 were clustered into the same group with KmBGL1 from *K*. *marxianus*, another yeast species closely related to *Clavispora* (Fig 8). Interestingly, β-glucosidase Ncbgl1 from *N*. *crassa* also fell in this group more closely related to BGL2. BGLs from yeast in general were distantly related to BGLs from *Trichoderma* and the bacterial BpBGLb (Fig 8). Other β-glucosidases from filamentous fungi, including *N*. *crassa* tested in this study did not appear to be closely related each other.

## Discussion

In this study, we cloned two new genes and characterized their encoding β-glucosidase BGL2 and BGL3, respectively, from *Clavispora* NRRL Y-50464. Through quantitative gene expression analysis and heterologous expression, we further confirmed the function of *BGL2* and *BGL3* genes utilizing cellobiose as a sole carbon source. Our research defined a family of at least three β-glucosidase encoding genes, including a recently characterized *BGL1*, in strain NRRL Y-50464. We also demonstrated the affinity and activity of BGL2 and BGL3 toward 14 oligosaccharides including cellobiose, cellopentaose, laminaribiose, and laminaripentaose, in addition to traditionally used chromogenic substrate assays. Both proteins showed high levels of tolerance to glucose and BGL2 also tolerated ethanol challenges up to 16%. In addition, these enzymes were active in the presence of fermentation inhibitors furfural and HMF. These results provided strong evidence to support the cellobiose hydrolytic function of strain Y-50464 as a potential candidate biocatalyst for low-cost cellulosic ethanol production using SSF.

The key function of β-glucosidase is to convert cellobiose into simple sugar glucose. Since most β-glucosidases are susceptible to high levels of glucose product inhibition, searching for glucose tolerant β-glucosidases has become a major challenge in lignocellulose-to-ethanol conversion [40, 41]. In this study, BGL2 and BGL3 both displayed an outstanding tolerance in the presence of high levels of glucose up to 2 M. Their performance on glucose product inhibition was superior to previously reported BGL1 and Novo188 as measured by *K*_*i*_ (Table 2). Strain Y-50464 was found to have other desirable characteristics including fast rate of cell growth and ethanol fermentation using SSF [24, 25]. Findings of glucose tolerance in this study partially explained the efficient utilization of cellobiose as a sole carbon source by this strain. While both BGL2 and BGL3 functioned well on glucose and cellobiose, their gene expression response to the two sugars appeared different. The expression level of *BGL2* was higher growing on glucose but showed a slightly transient lag before its recovery when shifted to cellobiose. In contrast, expression level of *BGL3* was relatively lower on glucose but increased significantly when shifted to cellobiose. In fact, BGL2 demonstrated significantly higher specific activity compared with that of BGL3. The lower levels of expression by *BGL2* than *BGL3* under these conditions were unexpected. It could reflect a robust native capability to utilize both glucose and cellobiose. The distinct amino acid sequence variations of BGL2 from BGL1 and BGL3 could be attributed to its outstanding functions. Sequence structure and function relationships of BGL2 is worth further studies to illustrate the mechanisms of its function. Both BGL2 and BGL3 also showed an acceptable activity toward a broad range of oligosaccharides. These enzymes were capable of not only hydrolyzing cellobiose but also functioned on aryl β-glucoside. Such results suggested BGL2 and BGL3 likely fall into the category of β-glucosidase with a broad substrate specificity.

While BGL3 was susceptible to a challenge of ethanol, BGL2 displayed increased activity in the presence of 4 to 12% ethanol. Inhibition or enhancement of enzyme activity by ethanol is commonly observed for β-glucosidases although many enzymes are inhibited by ethanol. The increased catalytic potential in the presence of ethanol has been attributed to the glycosyl transferase activity of some β-glucosidases [31, 42, 43]. Ethanol serves as an acceptor of a glycosyl intermediate to increase the reaction rate. Alternatively, the altered membrane permeability facilitates a ready access of the intracellular enzyme to the substrate. Since BGL3 was highly susceptible to ethanol, the functional mechanism of ethanol on the two enzymes is apparently different.

The two enzymes also responded differently in the presence of HgCl2 and EDTA. BGL2 activity was inhibited but BGL3 was not affected by these compounds. Inhibitions by these chemicals were previously observed for some β-glucosidases and thought to be due to the sensitivity of thiol group in the catalytic site of the enzyme [44, 45]. In general, both enzymes were not sensitive to other metal ions and chemicals including furfural and HMF. Furfural and HMF are common fermentation inhibitors derived from lignocellulose pretreatment. Strain Y-50464 was able to complete cellulose-to-ethanol conversion using SSF in the presence of furfural and HMF [10]. Findings in this work further confirmed additional tolerance of BGL2 and BGL3 to these inhibitors. On the other hand, we found that the activity of both BGL2 and BGL3 was inhibited by Cu^2+^ in this study. The inhibitory effect by copper has also been observed for other β-glucosidases [25, 44, 45]. It is possible that Cu^2+^ may hinder the substrate binding or inhibit the active site catalytic reaction. A severe inhibition by SDS was observed for both enzymes that essentially killed the reaction. Similar inhibition was previously reported for β-glucosidase suggesting that the active site was not dependent on divalent cations for enzyme activation [45]. The high levels of activity detected for both enzyme reactions in the presence of Fe^2+^ were consistently observed, however, the mode of the enhanced activity is currently unknown.

## Conclusion

The current study on gene cloning and characterization of two partially purified β-glucosidases BGL2 and BGL3 defined a β-glucosidase gene family with at least three members in this group, including a previously characterized BGL1 [25], from *Clavispora* NRRL Y-50049. BGL2 reported in this study showed the highest enzyme activity and strongest substrate affinity in this group that can be the major enzyme for cellobiose hydrolysis for this yeast strain. Active to a wide range of substrate and tolerance performance of BGL2 and BGL3, especially for the tolerance to glucose inhibition, are desirable characteristics required for high levels of solids load cellulosic ethanol production. Results of this study provided strong evidence to support the cellobiose hydrolysis function of NRRL Y-50464 and its potential as a candidate for lower cost cellulosic ethanol production. The concept of economic consolidated bioporcessing procedure in advanced biofuels production is widely accepted [46, 47]. Strain NRRL Y-50464 has demonstrated its high rate of ethanol conversion and titer levels from cellulosic materials by SSF. Results of this study further evidenced its β-glucosidase hydrolysis function and add new knowledge to understand the unique dual functions of this yeast strain. Outcomes of this research facilitate continued efforts toward the next-generation biocatalyst development for low-cost advanced biofuel production.

## Supporting Information

S1 Fig SDS-PAGE gels showing raw proteins from various elution samples for BGL2 (A) and BGL3 (B). (TIFF)
